# An Unusual Presentation of a “Gastric Mass”

**DOI:** 10.14309/crj.0000000000001740

**Published:** 2025-06-16

**Authors:** Madeline Washburn, Justin Bejcek, Anupama Ancha, Alexis Bejcek, Christopher Williams

**Affiliations:** 1 Department of Medicine, Division of Internal Medicine, Baylor Scott and White Medical Center, Temple, TX; 2 Department of Medical Education, Edward Via College of Osteopathic Medicine, Louisiana, Monroe, LA; 3 Department of Medicine, Division of Gastroenterology, Baylor Scott and White Medical Center, Temple, TX

**Keywords:** gastric band, bariatric surgery, gastric mass, gastrointestinal foreign body

## Abstract

Gastric bands have been used since the 1970s for weight loss. Owing to a rise in complications, this procedure has significantly decreased in popularity. Our study describes a 63-year-old woman with a history of nonadjustable gastric band placement 30 years ago who presented with an incidental imaging finding concerning for a gastric mass. Esophagoduodenoscopy revealed erosion of gastric band material through the gastric wall. Our case highlights the importance of endoscopy for assessment of abnormal imaging, including surgical complications in the differential for intraluminal masses, and obtaining a thorough surgical history.

## INTRODUCTION

In 1980, Dr. Marcel Molina placed his first nonadjustable gastric band (NAGB) using Dacron, a polyester fiber.^1,2^ This innovation provided patients with a noninvasive, reversible means to aid in weight loss by placing the band at the gastroesophageal junction, creating a small pouch to increase satiety. Gastric bands have decreased in popularity due to complications, including bleeding, infection, obstruction, perforation, and gastroesophageal reflux.^3,4^ Less frequently, patients may develop later-onset complications, including band slippage and erosion with band migration.^5–9^

Between 10% and 34% of lap band complications lead to reoperative procedures, complete removal, band adjustments, or other invasive bariatric surgeries.^10^ Removal methods typically involve open or laparoscopic surgery, although endoscopic removal has also been previously documented.^9^ This case presents a unique occurrence of NAGB erosion presenting as an intraluminal mass within a hiatal hernia, which was diagnosed on endoscopic assessment.

## CASE REPORT

**Figure 1.**
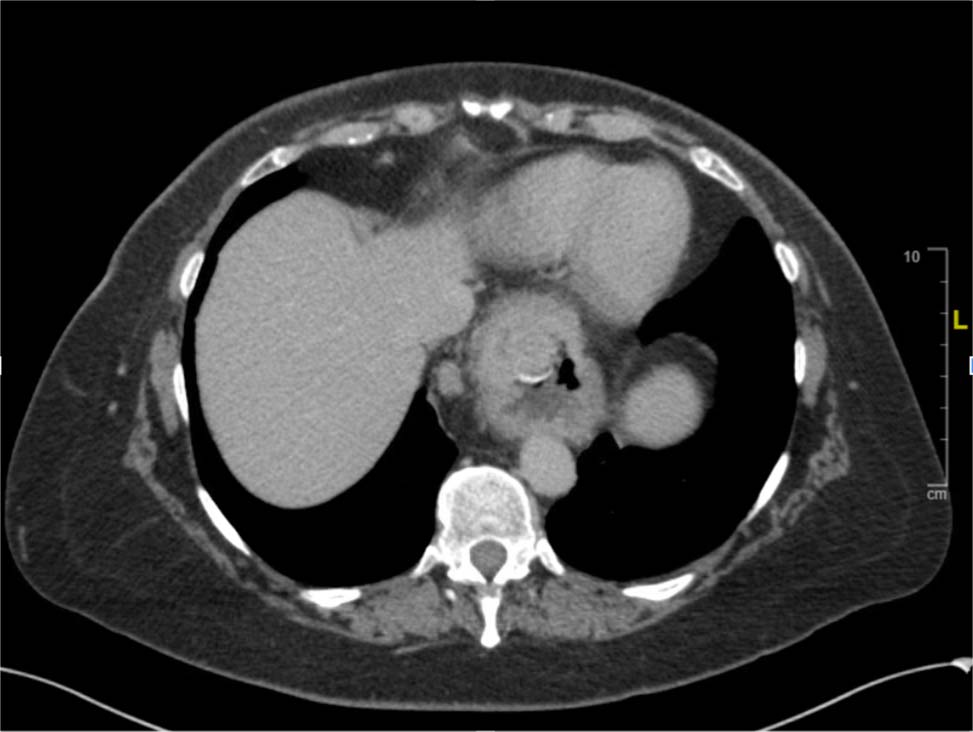
Computed tomography imaging with contrast demonstrating an intraluminal soft tissue lesion within the hiatal hernia measuring approximately 2.3 cm in transaxial dimension. There is an adjacent borderline enlarged paraesophageal lymph node measuring 9 mm.

A 63-year-old woman with a history of pulmonary embolism, hypothyroidism, and obesity status-post gastric lap band surgery 30 years ago presented to the gastroenterology clinic for evaluation of an incidental finding on abdominal imaging. She was following with urology for hematuria when a computed tomography urogram found a moderate-sized hiatal hernia with an intraluminal soft tissue gastric mass and adjacent enlarged paraoesophageal lymph nodes concerning for a malignancy (Figure 1). The patient denied any symptoms of dysphagia, epigastric pain, and melena. Her laboratory findings were largely unremarkable. She agreed to undergo esophagoduodenoscopy to investigate the findings and rule out malignancy.

**Figure 2.**
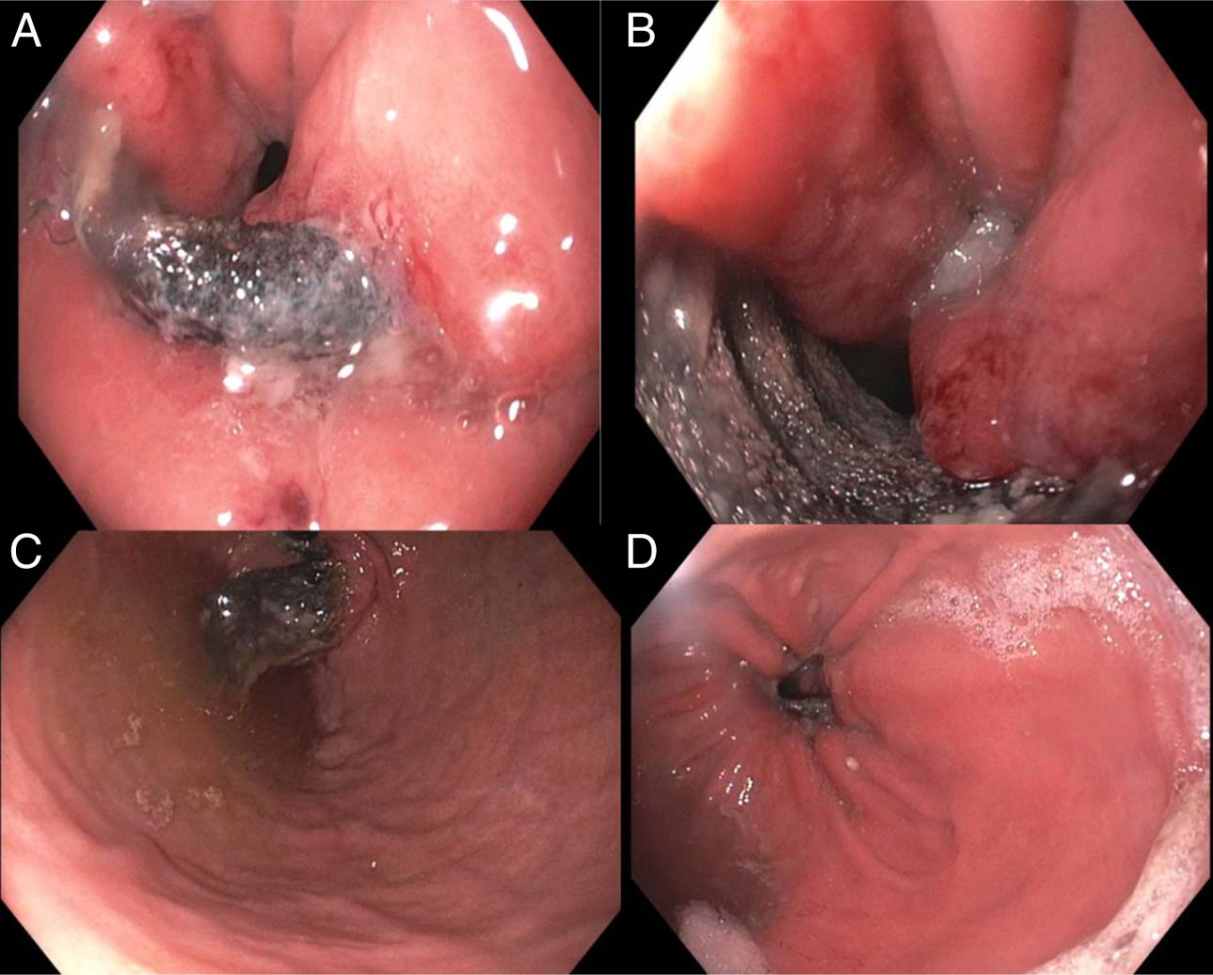
Endoscopic images of mesh-like material eroding through the gastric wall, occupying a large part of the diaphragmatic hiatus. (A and B) diaphragmatic hiatus; (C) gastric cardia in retroflexion; (D) hiatal hernia view from gastroesophageal (GE) junction.

During the procedure, the endoscopists discovered a mesh-like material eroding through the gastric wall, occupying a large part of the diaphragmatic hiatus (Figure 2). The foreign body material was consistent with the lap band which was placed 30 years ago to this evaluation. After shared decision making with the patient, she was seen by the bariatric surgery clinic. Further discussion revealed that, although initially successful, she slowly regained the weight she lost following her NAGB surgery, plus an additional 25 pounds. Because it was nonadjustable, she had no revisions to her gastric band since her surgery. She ultimately proceeded with laparoscopic removal of the eroded band, partial gastrectomy with Roux-en-Y reconstruction, and repair of the paraoesophageal hiatal hernia.

## DISCUSSION

The concept, design, and immediate outcomes demonstrated by the NAGBs were initially very favorable. However, due to a rise in complications, this procedure has significantly decreased in popularity.^1^ The inherent obstructive mechanism that led to its effectiveness was also noted to lead to serious long-term complications, as seen in our case.

Other banding procedures, such as laparoscopic adjustable gastric bands (LAGBs), became popular in the 1990s after the complication rates of the nonadjustable procedure became more apparent. However, it too came with short-term and long-term complications such as band migration and erosion.^11^ The LAGB has the additional risk of band fluid leakage and port-site infection, which is not a concern with the NAGB.^1,12^ Newer, less invasive weight loss procedures are now becoming more common, such as endoscopic sleeve gastroplasty. A recent study showed that patients with prior LAGB, though attenuated, still benefit from undergoing subsequent endoscopic sleeve gastroplasty.^13^

If complications such as band migration or erosion occur, there are both surgical and endoscopic options for removal. Adjustable lap bands also require a surgical component due to the presence of a subcutaneous port.^14^ To remove the bands endoscopically, the decision is multidisciplinary and requires surgical evaluation. In 2023, Manos et al demonstrated that endoscopic removal can be done; however, the timing of removal depends on percentage of band migration noted on first evaluation. If the band migrated more than 50% of its original diameter, it could be removed immediately. However, if it migrated <50%, either monitoring with reevaluation or stent placement to accelerate migration would be required.^15^ For our patient, the material eroded through her hernia making endoscopic removal more complicated. Given she already required repair of her paraoesophageal hiatal hernia, a surgical approach was indicated.

Nearly a decade ago, it was concluded that other methods of bariatric weight loss were preferred over banding. A study completed in 2006 by Suter et al demonstrated a 5-year fail rate of 40% and a 7-year success rate of only 43%.^16^ Their follow-up data were collected prospectively over 8 years postsurgery. Our patient's presentation over 30 years after her surgery shows that patients will continue to present with complications of banding procedures. Predisposing factors which can increase the risk of long-term complications include smoking and comorbidities such as diabetes, chronic kidney disease, and chronic steroid use. Other factors, such as medication noncompliance, poor follow-up, and poor diet and exercise habits can also lead to earlier complications.^17^ Our patient's delayed presentation and lack of complications is, in part, likely due to being a highly motivated never-smoker with good follow-up.

Although asymptomatic, our patient's inflammatory reaction to the Dacron band and the erosion that resulted were unfortunate complications that required intervention. Our case highlights the importance of using endoscopy for further assessment of abnormal imaging, including surgical complications in the differential diagnoses of intraluminal masses, and obtaining a thorough surgical history which includes interventions completed decades before evaluation.

## DISCLOSURES

Author contributions: M. Washburn: Drafting of manuscript and critical revision and is the article guarantor; J. Bejcek: Drafting of the manuscript; A. Ancha: Study conception, drafting of manuscript, and critical revision; A. Bejcek: Drafting of the manuscript and critical revision; C. Williams: Study conception, drafting of manuscript, and critical revision.

Financial disclosure: None to report.

Informed consent was obtained for this case report.

## ABBREVIATIONS:

LAGBs: laparoscopic adjustable gastric bands
NAGB: nonadjustable gastric band
